# Optic Disc, Macula, and Retinal Nerve Fiber Layer Measurements Obtained by OCT in Thyroid-Associated Ophthalmopathy

**DOI:** 10.1155/2016/9452687

**Published:** 2016-07-17

**Authors:** Osman Sayın, Volkan Yeter, Nurşen Arıtürk

**Affiliations:** ^1^Department of Ophthalmology, Beyhekim State Hospital, Konya, Turkey; ^2^Department of Ophthalmology, NEON Hospital, 24100 Erzincan, Turkey; ^3^Department of Ophthalmology, Ondokuz Mayıs University, Samsun, Turkey

## Abstract

*Aim*. To compare the measurements of retinal nerve fiber layer (RNFL), macula and optic disc parameters obtained by optical coherence tomography (OCT), and intraocular pressure (IOP) between the patients with thyroid-associated ophthalmopathy (TAO) and healthy controls.* Methods*. One hundred and thirty-two eyes of 66 patients with TAO and 72 eyes of 36 healthy controls were included in the study. Proptosis level was determined by Hertel exophthalmometer. Optic disc, peripapillary retinal nerve fiber layer, and macula parameters were measured by OCT. All measurements of the patients were compared with those of age- and sex-matched healthy controls.* Results.* No statistically significant difference was found between the patients with TAO and control group in terms of demographic characteristics (*P* > 0.05). Exophthalmometer measurements and IOP were higher in TAO group (*P* < 0.05). Mean macula thicknesses in TAO and control groups were 239.3 ± 29.8 *μ*m and 246.6 ± 31.8 *μ*m, respectively, and the difference between the groups was statistically significant (*P* = 0.000). TAO group had thinner inferior RNFL thickness and macular thicknesses (superior, inferior, temporal, and nasal) and higher disc area and C/D ratio when compared with the control group (*P* < 0.05).* Conclusion.* IOP, disc area, and C/D area ratio were higher in the patients with TAO and the thicknesses of macula and inferior RNFL were thinner when compared with healthy controls. This trial is registered with registration number at clinicaltrials.gov NCT02766660.

## 1. Introduction

Thyroid-associated ophthalmopathy (TAO) is autoimmune inflammation of orbital tissues. It is accompanied by inflammatory cellular infiltration with lymphocytes, plasma cells, macrophages, and mast cells of interstitial tissues, orbital fat, and lacrimal glands associated with accumulation of glycosaminoglycans and retention of fluid. This causes increase in the volume of orbital contents and secondary elevation of intraorbital pressure [1, 2]. Increased intraorbital pressure and the swelling of the extraocular muscles at the apex cause compressive optic neuropathy and visual loss [3].

It was shown that optic disc function may be affected in TAO patients without obvious optic neuropathy and extraocular muscle swelling [4–6]. So some diagnostic and follow-up tests were previously used to evaluate the visual function of the patients with TAO, such as visual field test, contrast sensitivity test, visual evoked potential, and color sensation test. Optical coherence tomography (OCT) is an alternative and noninvasive test to evaluate the macula and the optic disc. Previously, only three studies investigating retinal nerve fiber layer thickness and optic disc morphology measured by OCT in TAO patients were reported [7–9] and there is no study evaluating macular thickness by OCT in TAO patients.

In this study, we have examined retinal nerve fiber thickness, macula, and optic nerve head by OCT in TAO patients and compared the measurements of TAO patients with those of age- and sex-matched healthy subjects.

## 2. Materials and Methods

Sixty-six patients with ophthalmopathy who were diagnosed as thyroid pathology by the Department of Endocrinology, Ondokuz Mayıs University, Samsun, Turkey, and were sent to ophthalmology clinic for eye involvement and 36 healthy volunteers who formed the control group were included in our study. Written informed consents were obtained from all subjects. The study was approved by the Human Research Ethics Committee of Ondokuz Mayıs University, Samsun, Turkey, and was conducted in accordance with the tenets of the Declaration of Helsinki. The control group was chosen among healthy volunteers who did not have any eye pathology, who had a 20/20 vision (corrected or uncorrected), and who were age- and sex-matched with the patient group. In both groups, those who had significant sight impairment, high myopia (<−5 D), high hyperopia (>+3 D), optic disc anomaly, vitreoretinal interface disease, vascular and degenerative retinal diseases, cornea or lens opacity, ocular surgery history, glaucoma, neurological diseases that can affect the visual field, history of trauma, amblyopia, diplopia, keratitis, and history of topical or systemic steroid use were not included in the study.

Medical history of all the cases was taken, and the cases underwent a detailed ophthalmologic examination which included best-corrected visual acuity, color vision, biomicroscopic anterior segment examination, and intraocular pressure measurement in sitting position with the Goldmann Applanation Tonometry and dilated fundus examination. The proptosis levels of all cases included in the study were measured with Hertel exophthalmometer. Ophthalmic involvement of the patients with thyroid-associated ophthalmopathy was scored according to the NOSPECS classification.

The patients in the research group were grouped according to their measurements of Hertel exophthalmometer. The first group was those between 21 and 23 mm, the second group was those >23 mm, and the third group was those with a difference of more than 2 mm between the eyes. The measurements were compared among the three groups and with the control group. Optical coherence tomography (Stratus OCT; Carl Zeiss Meditec, Inc., Dublin, California, USA) (software version 4.4) was employed to measure the eyes of TAO patients and control group. The measurements of these groups were compared. The differences between the right and left eyes were examined and they were separately compared with the control group. Whether there was correlation between the period of time that passed between the time when the diagnosis of thyroid was made and the time ophthalmopathy was seen, NOSPECS, clinical activity scores, and values were investigated.

The data obtained from the study was transferred to computer and analyzed with SPSS 15.0 (SPSS, Chicago, II, USA) package program. The normality test in patient and control groups was checked and paired samples *t*-test was used for the values which were normally distributed while the Wilcoxon Rank test was used for the values which were not normally distributed. The values with *P* < 0.05 were considered to be statistically significant. In the comparison of the patient and control groups, Student's *t*-test was used for data with parametric distribution while Mann-Whitney* U* test was used for data with no parametric distribution.

## 3. Results

One hundred and thirty-two eyes of 66 patients who were diagnosed as thyroid ophthalmopathy and the 72 eyes of 36 healthy volunteers were included in the study. Mean ages were 40.1 ± 12.6 years (range: 12–73 years) and 38.3 ± 9.7 (range: 13–69 years) for the TAO and control groups, respectively, while no significant difference was found between the two groups (*P* > 0.05). Forty-three (65.2%) of the TAO group were female while 23 (34.8%) were male and 25 (69.4%) of the control group were female while 11 (30.6%) were male. No statistically significant differences were found between the two groups in terms of demographic features (*P* > 0.05). Mean Hertel exophthalmometer values were 19.1 ± 3.0 mm and 15.2 ± 1.2 mm for the TAO and control group, respectively, and the difference was statistically significant (*P* < 0.05). All of the patients had bilateral TAO and the NOSPECS score of the patients was 2.4 ± 0.9 (range: 1–5).

Mean intraocular pressure was 14.3 ± 2.7 mmHg for the TAO patients. The averages were 14.3 ± 2.6 mmHg and 14.3 ± 2.7 mmHg for the right and the left eye, respectively (*P* > 0.05). Mean intraocular pressure was 12.9 ± 1.9 mmHg for the control group. Mean IOP was 13.0 ± 1.9 mmHg and 12.7 ± 2.0 mmHg for the right and the left eye of control group, respectively (*P* > 0.05). When the patient group with TAO and the control group were compared for IOP, the difference between the mean IOP values was statistically significant (*P* = 0.011). In addition, the difference was also statistically significant when the right and left eyes of the TAO and the control group were compared with each other (*P* < 0.05) (Table 1).

Mean temporal and inferior RNFL thicknesses of the TAO group were 72.1 ± 15.4 *μ*m and 124.8 ± 14.8 *μ*m, respectively, for the right eye while they were 65.1 ± 10.7 *μ*m and 116.5 ± 17.8 *μ*m, respectively, for the left eye and the difference between the left and right eyes was statistically significant (*P* < 0.05). Mean inferior RNFL thicknesses of the control group were 130.6 ± 20.4 *μ*m and 123.3 ± 18.3 *μ*m, respectively, for the right and the left eye and the difference was statistically significant (*P* = 0.021). No significant difference was found between TAO patients and the controls for the other RNFL values. No significant difference in RNFL thicknesses for both right and left eyes separately was found between the groups (Table 1). When both eyes of the patients in the control and patient groups were assessed together for RNFL, only the difference between the inferior RNFL values of the patient and the control group was found to be statistically significant. Mean inferior RNFL thicknesses were 120.7 ± 16.8 *μ*m and 127.0 ± 19.6 *μ*m for the patient and the control groups, respectively (*P* < 0.05) (Table 2).

Only mean superior macula thickness of the patient group showed significant difference between the right and left eyes (*P* = 0.031) although there was no difference between the eyes for exophthalmometer measurements (*P* > 0.05). Mean of superior macula thicknesses in TAO group was 254.0 ± 14.2 *μ*m and 256.1 ± 14.5 *μ*m for the right and left eye, respectively. No significant difference was found between the right and left eye for any macula parameters in control group. The right mean macula thickness and central, temporal, superior, and nasal quadrant thicknesses of the patient group were found to have significant thinning when compared with the right eye of the control group (*P* < 0.05) (Table 1). In the left eye, this thinning was seen in mean macula thickness and central and temporal macula thicknesses (*P* < 0.05). When both eyes of the patients in the control and patient groups were assessed together, a thinning was observed in the macula thicknesses of all quadrants in the study group (*P* < 0.05) (Table 2).

In terms of optic disc, no significant difference was found in the values of the patient and control groups. When the right disc area and the left disc area were checked, a significant increase was seen in the patient group (*P* < 0.05) (Table 1). No significant difference was found between the right and the left eyes in terms of other disc values. When both eyes of the patients in the control and TAO groups were assessed together, a statistically significant increase was found in the patient group in terms of disc area and C/D ratio (*P* < 0.05) (Table 2).

A total of 47 patients who had proptosis values of 21 mm and over 21 mm were grouped in three and compared with the control group. In the classification of the patient group with TAO, there were 33 eyes in the first group (21–23 mm), 10 eyes in the second group (>23 mm), and 4 eyes in the third group (>2 mm difference). When the intraocular pressure values were analyzed, mean IOP measurements were 15.1 ± 2.4 mmHg in the first group, 16.3 ± 2.0 mmHg in the second group, and 17.0 ± 4.5 mmHg in the third group and no statistically significant differences were found between the groups (*P* > 0.05). The mean IOP measurements of the control group and the three groups were 12.9 ± 1.9 mmHg and 15.5 ± 2.5 mmHg, respectively, and the difference between them was statistically significant (*P* = 0.000) (Table 2). In the comparison of the three groups separately with the control group in terms of IOP values, the differences were found to be statistically significant (*P* = 0.000) (Table 3). In the comparison of TAO groups with one another in terms of IOP values, no statistically significant difference was found (*P* > 0.05). When the correlation between IOP and proptosis values was analyzed, no important correlation was found in Group 1. Group 2 was found to have a moderate positive correlation (*r* = 0.68, *P* = 0.27) while group 3 was found to have a poor positive correlation (*r* = 0.19, *P* > 0.05).

In the comparison of the three groups (47 eyes) with the control group (72 eyes), inferior RNFL thicknesses were 120.0 ± 17.5 *μ*m and 127.0 ± 19.6 *μ*m in the TAO and control group, respectively (*P* = 0.05). In the comparison of macula values, mean and central macula thicknesses of the patient group were found to be thinner than the control group (*P* < 0.05). In the comparison of disc parameters, disc area and C/D ratio were found to be greater in the patient group when compared with the control group (*P* < 0.05). No significant difference was found in terms of other RNFL, macula, and disc values (*P* > 0.05).

When Groups 1 and 2 were compared, vertical C/D ratio was found to be 0.47 ± 0.15 and 0.33 ± 0.18, respectively, and the difference between them was found to be statistically significant (*P* = 0.016). Disc areas were 2.66 ± 0.48 mm^2^ and 2.15 ± 0.53 mm^2^, respectively, and the difference was found to be statistically significant (*P* = 0.007). When the 10 eyes in the second group which had Hertel exophthalmometer values of >23 mm were compared with the control group, only the thinning in the rim area was found to be significant (*P* = 0.036) (Table 3). No statistically significant difference was found for the other values (*P* > 0.05). When Groups 1 and 3 and Groups 2 and 3 were compared in terms of RNFL, macula, and disc values, no statistically significant difference was found.

When the correlations between the time that passes between the diagnosis of thyroid and the occurrence of ophthalmopathy (15.4 ± 41.7 months), NOSPECS and clinical activity score, and the OCT measurements were analyzed, statistically insignificant and poor correlation was found (*r* between −0.05 and 0.30, *P* > 0.05).

## 4. Discussion

Thyroid-associated ophthalmopathy (TAO) is a progressive and organ specific autoimmune disease. In TAO patients, one of the most important circumstances that need to be assessed before the decision of treatment is the activity of the disease. TAO starts with an active progressive phase and then it moves to the inactive phase. Clinical activity scoring (CAS) system is used to find out the disease activity [10]. In our study, the mean CAS of the TAO patient group was 1.4 ± 1.1. Although most of the patients were at early phase of the disease in the study, we found that mean inferior RNFL was significantly thinner in the TAO group when compared with the controls and there was no statistically significant difference for mean RNFL thickness between groups. Wei et al. studied 151 eyes of 76 TAO patients with eye movement restriction in any 1 of 4 primary directions [7]. They reported that the cross-sectional area of all rectus muscles obtained by computerized tomography was not significantly correlated with logMAR or proptosis, nor with the total thickness of the peripapillary nerve fiber layer (RNFL), and the sum of all quadrants of the peripapillary nerve fiber layer thickness was not significantly correlated with other visual function parameters [7]. Wei et al. did not use the RNFL parameters (superior, inferior, temporal, and nasal) separately for the analysis in their study and they used the sum of all quadrants of RNFL thickness, and they performed the measurements in Chinese people. It was known that normative data of RNFL measurements may show some varieties in different races. So we measured RNFL thicknesses of age- and sex-matched healthy Caucasians for the comparison in our study. In some cases, optic nerve stretching without muscle involvement may cause visual impairment [6]. There are some cases of dysthyroid optic neuropathy with progressive visual field loss, but with normal-sized or minimally enlarged extraocular muscles [5].

Inferior and temporal RNFL values of the left eye in TAO group and inferior RNFL values of the left eye in control group were found significantly thinner than the right eye. Although there were no statistically significant differences between the right and the left eyes in terms of proptosis and IOP values, it was thought that the difference in RNFL thickness within the groups could be resulting from the fact that TAO affected eyes in the same patient in different degrees because of the factors other than IOP and proptosis. Although TAO frequently shows bilateral involvement, studies have shown that the involvement can also be asymmetric. It has been stated that orbital structural differences may cause asymmetric blood flow, lymphatic drainage, or tissue enlargement and, as a result of this, bilateral but asymmetric involvement can be seen [11, 12].

It was reported that there was a statistically significant difference between right and left eyes in terms of inferior RNFL thickness in healthy people [13, 14]. Similarly, a statistically significant difference was found between inferior RNFL thicknesses in the control group and this difference was thought to be physiological since none of the patients in the group had ophthalmologic diseases.

There are studies which show that glaucoma prevalence in patients with thyroid pathology is higher when compared with the normal population [15]. Some of the studies show increases in IOP while some show no differences [16, 17]. It was shown that when the patients with hypothyroidism were treated, a decrease in IOP may be observed after the treatment and there was a possible effect of thyroid disease on IOP [18]. Macula thicknesses may be affected by the changes in IOP. In patients with glaucoma, ganglion cell damage was shown in macula [19, 20]. In Sesar et al.'s study [21], as a result of the decrease in IOP after glaucoma filtration surgery, macular thicknesses increase within a month. They thought that this occurred as a result of the physiological response of retina to the sudden decrease in IOP. Tan et al. [22] found that the macula thicknesses in perimetric glaucoma patients were thinner. In their study, Arvanitaki et al. [23] compared patients with a suspicion of glaucoma who have early manifest glaucoma and nontreated IOP averages of 17 mmHg. They found that although there was no peripapillary RNFL change, there was a significant thinning in the superior, inferior, nasal, and temporal macular thicknesses of the patient group when compared with the control group. Another study showed that the thinning was seen in inferior macular thickness [24]. It has been suggested that the localized thinning in inferior and superior macula thicknesses might reflect the retinotopic distribution of the affected ganglion cells. In our study, thinning was observed in all segments of the macula of the TAO group when compared with the control group. It was thought that the decreases in the macula thickness could be due to the higher values of IOP and proptosis in TAO groups when compared with the control group. It was also thought that optic nerve damage could occur as a result of fixed orbital volume despite the enlargement of retrobulber connective tissue, and as a result of this ganglion cell damage could occur which could first be seen with the thinning in macular thicknesses. Nevertheless, it may be thought that the higher levels of IOP in TAO groups, even in normal limits, could cause ganglion cell damage and macular thinning, and the mechanical stress caused by the increase in IOP could affect the macula before peripapillary nerve fiber damage because of a malady in retina inner dynamics.

Hypoxia and ischemia caused by the edema in the orbital soft tissues were thought to cause retinal changes before optic nerve compression developed. There are studies in literature which show thinning in the macula thicknesses in parallel with the severity of glaucoma and it was thought that macular ganglion cell complex thickness could be a more accurate indicator than peripapillary nerve fiber thicknesses in early glaucoma cases and this is used in monitoring the progression of the disease [25]. It may be thought that the thinning of macular thickness in our study may be caused by thinning in macular ganglion complex. Macular ganglion cell complex thickness should be investigated by spectral-domain OCT in thyroid ophthalmopathy patients to detect the level of the thinning and the effect of the disease on the macular ganglion complex.

Poostchi et al. [26] found an expansion in the disc area as a result of IOP increase in patients who did not have glaucoma. In our study, it was thought that the increase in IOP could be responsible for the expansion in disc area, C/D ratio and vertical and horizontal C/D ratio, and the thinning in the rim area.

When the eyes of the patient group and the control group were compared, the mean IOP in both eyes of the TAO group was found significantly higher. Similarly, Sen et al. [9] reported that IOP was higher in the TAO patients and mean RNFL thickness of TAO patients was thinner than those of control group. Even if relatively milder cases according to the NOSPECS classification were included in our study, the increase in the IOP of the patient group may be caused by the increased volume of intraorbital structures. Chu et al. [27] found that the plasma ET-1 levels were higher than normal levels in thyroid hormone disorders caused by Graves' disease. ET-1, which is a strong vasoconstrictor, can show a decreasing effect on the blood flow of optic nerve head. Peripapillary RNFL may be influenced by ischemic situations which are caused by the vasoconstrictor effect of ET-1. Even in the absence of glaucoma, this vascular deficiency in peripapillary area and retina can trigger some changes in the optic nerve head and macula [28].

Forte et al. [8] found that there was a decrease in RNFL in patients with Graves' ophthalmopathy and ocular hypertension when compared with the control group and this decrease was found to be significantly higher in inferior and superior quadrants. In our study, a significant thinning was found in inferior RNFL thickness in TAO patients when compared with healthy controls.

## 5. Conclusion

In the study, we found that the macula and retinal nerve fiber layer thicknesses were thinner and intraocular pressure, disc area, and C/D ratio were higher in the patients with thyroid-associated ophthalmopathy. The study is the first one that shows the effects of thyroid ophthalmopathy on the macular thicknesses obtained by time-domain OCT. The patients in our study were in early phases and OCT parameters were not monitored for a long time. Further longitudinal follow-up studies performed by spectral OCT with homogenous sample are needed to see the long term effect of the disease on the RNFL thickness, optic disc, and macula.

## Figures and Tables

**Table 1:** Comparison of the parameters between the patients with thyroid-associated ophthalmopathy (TAO) and control group according to side of the eyes.

Parameters	Right eye		*P*	Left eye		*P*
	TAO	Control		TAO	Control	
Hertel exophthalmometer (mm)	19.1 ± 3.0	15.2 ± 1.1	0.000^*∗*^	19.2 ± 3.0	15.1 ± 1.3	0.000^*∗*^
IOP (mmHg)	14.3 ± 2.6	13.0 ± 1.9	0.016^*∗*^	14.3 ± 2.7	12.7 ± 2.0	0.003^*∗*^
Mean RNFL (*µ*m)	99.9 ± 29.3	102.8 ± 29.7	>0.05	96.7 ± 29.4	100.7 ± 31.1	>0.05
Nasal RNFL (*µ*m)	79.2 ± 16.4	83.7 ± 16.2	>0.05	80.0 ± 16.1	81.5 ± 16.5	>0.05
Inferior RNFL (*µ*m)	124.8 ± 14.8	130.6 ± 20.4	>0.05	116.5 ± 17.8	123.3 ± 18.3	>0.05
Superior RNFL (*µ*m)	123.4 ± 18.3	123.9 ± 13.0	>0.05	125.3 ± 17.1	128.9 ± 18.5	>0.05
Temporal RNFL (*µ*m)	72.1 ± 15.4	73.0 ± 14.4	>0.05	65.1 ± 10.7	69.0 ± 16.1	>0.05
Mean macula thickness (*µ*m)	238.6 ± 29.7	245.6 ± 27.9	0.010^*∗*^	240.0 ± 30.0	247.6 ± 35.3	0.011^*∗*^
Central macula thickness (*µ*m)	190 ± 23.6	203.3 ± 31.1	0.018^*∗*^	191.7 ± 23.9	203.9 ± 27.3	0.025^*∗*^
Nasal macula thickness (*µ*m)	260.6 ± 13.7	266.1 ± 12.0	0.044^*∗*^	262.0 ± 15.4	264.5 ± 18.5	>0.05
Inferior macula thickness (*µ*m)	248.9 ± 11.4	252.9 ± 10.0	>0.05	249.8 ± 12.2	253.9 ± 10.2	>0.05
Superior macula thickness (*µ*m)	254.0 ± 14.2	260.0 ± 10.0	0.017^*∗*^	256.1 ± 14.5	261.3 ± 11.4	>0.05
Temporal macula thickness (*µ*m)	239.9 ± 12.5	245.1 ± 10.6	0.021^*∗*^	240.6 ± 13.4	254.6 ± 50.2	0.016^*∗*^
C/D ratio (horizontal)	0.49 ± 0.17	0.44 ± 0.16	>0.05	0.48 ± 0.17	0.44 ± 0.18	>0.05
C/D ratio (vertical)	0.42 ± 0.14	0.39 ± 0.14	>0.05	0.42 ± 0.15	0.39 ± 0.16	>0.05
Disc area (mm^2^)	2.56 ± 0.49	2.31 ± 0.34	0.010^*∗*^	2.61 ± 0.51	2.30 ± 0.35	0.002^*∗*^
Rim area (mm^2^)	1.85 ± 0.52	1.82 ± 0.41	>0.05	1.94 ± 0.51	1.80 ± 0.45	>0.05
C/D ratio	0.26 ± 0.14	0.20 ± 0.13	>0.05	0.24 ± 0.13	0.21 ± 0.13	>0.05

Data is demonstrated as mean ± standard deviation.

RNFL: retinal nerve fiber layer.

IOP: intraocular pressure.

^*∗*^Statistical significance.

**Table 2:** Comparison of IOP and OCT parameters between the patients with thyroid-associated ophthalmopathy (TAO) and control group.

Parameters	TAO (*n* = 132 eyes)	Control (*n* = 72 eyes)	*P*
IOP (mmHg)	15.5 ± 2.5	12.9 ± 1.9	0.000^*∗*^
Mean RNFL (*µ*m)	98.3 ± 29.4	101.7 ± 30.4	>0.05
Nasal RNFL (*µ*m)	79.6 ± 16.2	82.6 ± 16.3	>0.05
Inferior RNFL (*µ*m)	120.7 ± 16.8	127.0 ± 19.6	0.043^*∗*^
Superior RNFL (*µ*m)	124.3 ± 17.7	126.4 ± 16.1	>0.05
Temporal RNFL (*µ*m)	68.6 ± 13.7	71.0 ± 15.3	>0.05
Mean macula thickness (*µ*m)	239.3 ± 29.8	246.6 ± 31.8	0.000^*∗*^
Central macula thickness (*µ*m)	190.9 ± 23.7	203.6 ± 29.1	0.003^*∗*^
Nasal macula thickness (*µ*m)	261.3 ± 14.5	265.2 ± 15.5	0.021^*∗*^
Inferior macula thickness (*µ*m)	249.4 ± 11.8	253.4 ± 10.0	0.016^*∗*^
Superior macula thickness (*µ*m)	255.1 ± 14.4	260.9 ± 10.6	0.003^*∗*^
Temporal macula thickness (*µ*m)	240.0 ± 12.9	249.9 ± 36.4	0.001^*∗*^
C/D ratio (horizontal)	0.48 ± 0.17	0.44 ± 0.17	>0.05
C/D ratio (vertical)	0.42 ± 0.14	0.39 ± 0.15	>0.05
Disc area (mm^2^)	2.58 ± 0.50	2.31 ± 0.34	0.000^*∗*^
Rim area (mm^2^)	1.89 ± 0.52	1.81 ± 0.43	>0.05
C/D ratio	0.25 ± 0.14	0.21 ± 0.13	0.030^*∗*^

Data is demonstrated as mean ± standard deviation.

IOP: intraocular pressure.

RNFL: retinal nerve fiber layer.

^*∗*^Statistical significance.

**Table 3:** Comparison of OCT parameters and IOP between control group and the patients with thyroid-associated ophthalmopathy based on Hertel exophthalmometer values.

Parameters	Control	Group 1 (*n* = 33)	*P*	Group 2 (*n* = 10)	*P*	Group 3 (*n* = 4)	*P*
IOP (mmHg)	12.9 ± 1.9	15.1 ± 2.4	0.000^*∗*^	16.3 ± 2.0	0.000^*∗*^	17.0 ± 4.5	0.000^*∗*^
Mean macula thickness (*µ*m)	246.6 ± 31.8	239.5 ± 32.9	0.019^*∗*^	240.8 ± 30.1	>0.05	246.9 ± 26.7	>0.05
Central macula thickness (*µ*m)	203.6 ± 29.1	184.5 ± 25.5	0.002^*∗*^	193.4 ± 18.7	>0.05	202.7 ± 12.1	>0.05
C/D ratio (vertical)	0.39 ± 0.15	0.47 ± 0.15	0.013^*∗*^	0.33 ± 0.18	>0.05	0.43 ± 0.10	>0.05
Disc area (mm^2^)	2.31 ± 0.34	2.66 ± 0.48	0.000^*∗*^	2.15 ± 0.53	>0.05	2.57 ± 0.14	>0.05
Rim area (mm^2^)	1.81 ± 0.43	1.83 ± 0.61	>0.05	1.49 ± 0.52	0.036^*∗*^	2.00 ± 0.22	>0.05
C/D ratio	0.21 ± 0.13	0.31 ± 0.17	0.003^*∗*^	0.24 ± 0.16	>0.05	0.21 ± 0.09	>0.05

Data is demonstrated as mean ± standard deviation.

Group 1: measurement of exophthalmometer between 21 and 23 mm; Group 2: >23 mm; Group 3: those with a difference of more than 2 mm between the eyes.

IOP: intraocular pressure.

^*∗*^Statistical significance.
